# AGREE II Quality Assessment of National and International Clinical Practice Guidelines on Prostate Cancer Management by the OPTIMA Consortium

**DOI:** 10.1016/j.euros.2024.10.020

**Published:** 2024-11-12

**Authors:** Vasileios Sakalis, Yagnaseni Bhattacharya, Katharina Beyer, Charlotte Murray, Emma Jane Smith, Peter-Paul M. Willemse, Giorgio Gandaglia, Romain Boissier, Angelika Borkowetz, Saeed Dabestani, Renee C.A. Leenen, Antoni Vilaseca, Gianluca Maresca, Jeremy Teoh, Juan Gómez Rivas, Pawel Rajwa, Michael Lardas, Nikolas Grivas, Thomas Van den Broeck, Benjamin Pradere, Natasha Schouten, Zafer Tandogdu, Susan Evans-Axelsson, Steven Maclennan, Marlene Thomas, Alberto Briganti, Anders Bjartell, Phil Cornford, Hagen Kruger, James N’Dow, Monique J. Roobol, Muhammad Imran Omar, OPTIMA Consortium

**Affiliations:** a Hippokrateion General Hospital of Thessaloniki, Urology, Thessaloniki, Greece; b Division of Medical and Dental Education, Aberdeen Medical School, Aberdeen, UK; c Department of Urology, Erasmus MC Cancer Institute, Erasmus University Medical Center, Rotterdam, The Netherlands; d Academic Urology Unit, University of Aberdeen, Aberdeen, Scotland, UK; e European Association of Urology Guidelines Office, Arnhem, The Netherlands; f Department of Urology, Cancer Center, University Medical Center Utrecht, Utrecht, The Netherlands; g Department of Urology and Division of Experimental Oncology, Urological Research Institute, Vita-Salute San Raffaele University, IRCCS San Raffaele Scientific Institute, Milan, Italy; h Department of Urology and Renal transplantation, APHM, Aix-Marseille Université, Marseille, France; i Department of Urology, Technische Universität Dresden, Dresden, Germany; j Department of Urology, Kristianstad Central Hospital, Region Skane, Kristianstad, Sweden; k Department of Translational Medicine, Section Division of Urological Cancers, Lund University, Lund, Sweden; l Servicio de Urología, Hospital Clínic de Barcelona, Barcelona, Spain; m Department of Urology, Aberdeen Royal Infirmary, Aberdeen, Scotland, UK; n S.H. Ho Urology Centre, Department of Surgery, The Chinese University of Hong Kong, Hong Kong, China; o Department of Urology, Hospital Clinico San Carlos, Madrid, Spain; p Department of Urology, Comprehensive Cancer Center, Medical University of Vienna, Vienna, Austria; q Department of Urology, Medical University of Silesia, Zabrze, Poland; r Department of Urology, Metropolitan General Hospital, Athens, Greece; s Department of Urology, Netherlands Cancer Institute, Amsterdam, The Netherlands; t Department of Urology, University Hospitals Leuven, Leuven, Belgium; u Department of Urology, La Croix Du Sud Hospital, Quint Fonsegrives, France; v Department of Urology, University College London Hospitals, London, UK; w Bayer AG, Berlin, Germany; x Roche, Basel, Switzerland; y Department of Translational Medicine, Lund University, Lund, Sweden; z Department of Urology, Liverpool University Hospitals Foundation Trust, Liverpool, UK; aa Medical Affairs, Pfizer, Berlin, Germany

**Keywords:** Prostate cancer, Clinical practice guidelines, AGREE II, Recommendations

## Abstract

**Background and objective:**

Clinical practice guidelines for prostate cancer (PCa) are a valuable resource for everyday clinical practice. The clinical practice guidelines and recommendations produced by various societies should demonstrate a considerable level of consistency in terms of quality, regardless of the society that developed these given the common evidence base. However, to date, no study has assessed the quality of PCa clinical practice guidelines. As part of the Optimal Treatment for Patients with Solid Tumours in Europe Through Artificial intelligence (OPTIMA) project, we evaluated the quality of the most frequently used national and international clinical practice guidelines for PCa using the Appraisal of Guidelines for Research & Evaluation II (AGREE II) tool.

**Methods:**

The quality of the identified clinical practice guidelines was assessed independently by two assessors using the AGREE II tool. The AGREE II tool comprises 23 different items organised into six domains, rated on a 7-point scale (1: strongly disagree to 7: strongly agree). The total score of the appraisal was the mean value of the two assessments. The agreement between assessors’ scores was calculated using the interclass correlation coefficient (ICC). Four key recommendations were compared among the included clinical practice guidelines to assess consistency.

**Key findings and limitations:**

Sixteen clinical practice guidelines were assessed using their latest available version (cut-off April 2024). The European Association of Urology, S3LL PCa, Belgian Health Care Knowledge Centre, National Comprehensive Cancer Network, and Prostatacancer—Nationellt vårdprogram guidelines received the highest overall scores with a mean domain score of 82.4% (range: 75.5–88.3%). The de l’Association Française d’Urologie (AFU), American Urological Association, and National Institute for Health and Care Excellence received a mean domain score of 77.6% (range: 73.7–84.0%). Below average were the European Society for Medical Oncology, localised (L) and systemic (S) CPPC American Society of Clinical Oncology, and Nederlandse Vereniging voor Urologie (NVU) with a mean domain score of 58.4% (range: 43.5–76.3%). The reasons for scoring below average included the following: inadequate information about the methodology applied, limited scope of the guideline, and limited patient engagement. The highest inter-rater variability was observed in NVU (ICC: 0.58) and the lowest in AFU-L (ICC: 0.84). When examining the scores of each domain, “clarity of presentation” (domain 4) achieved the highest score with a mean of 86.9% ± 12.6%. The domain with the lowest score was applicability (domain 5), with a mean of 48.3% ± 24.8%. The ICC was calculated to be 0.72 (±0.08).

**Conclusions and clinical implications:**

This is the first study in which a comprehensive quality assessment of the majority of international and national clinical practice guidelines was undertaken, and the key recommendations were compared to assess consistency. Our study shows that the majority of international and national clinical practice guidelines demonstrate high-quality standards when assessed using the AGREE II evaluation tool. The clinical practice guidelines that did not meet the expected standards could be improved by adopting several key recommendations outlined by our study.

**Patient summary:**

The OPTIMA project used the Appraisal of Guidelines for Research & Evaluation II (AGREE II) tool to evaluate the quality of 16 commonly used national and international clinical practice guidelines for prostate cancer. While some of these international and national clinical practice guidelines received the highest score, few guidelines scored below average due to methodological deficiencies and limited patient engagement. These findings highlight the need for a standardised process to ensure high-quality, consistent guidelines across practices.

## Take Home Message

This is the first study in which a comprehensive quality assessment of the majority of international and national clinical practice guidelines (CPGs) was undertaken, and the key recommendations were compared to assess consistency. Our study shows that the majority of international and national CPGs demonstrate high-quality standards when assessed using the Appraisal of Guidelines for Research & Evaluation II evaluation tool. The CPGs that did not meet the expected standards could be improved by adopting several key recommendations outlined by our study.

## 1 Introduction

Prostate cancer (PCa) is the most prevalent type of cancer affecting men, accounting for 24% of all cancer diagnoses in Europe [1]. Over the past decade, management of PCa has advanced significantly with the emergence of new diagnostic and therapeutic modalities. Physicians frequently refer to international or national guidelines to guide their practice. Therefore, clinical practice guidelines should be updated constantly and should provide recommendations based on the available evidence.

Prestigious organisations, such as the European Association of Urology (EAU), National Institute for Health and Care Excellence (NICE), or American Urological Association (AUA), formulate clinical practice guidelines according to a specific methodology. These internationally recognised clinical practice guidelines provide the latest evidence and recommendations on the diagnosis, treatment, and follow-up of patients with PCa. Hence, it is expected that clinical practice guidelines are largely concordant and nonconflicting, aiming to provide guidance for internationally standardised care.

The existence of multiple clinical practice guidelines worldwide could be one factor among others that partially explains variations in PCa management. Identifying the areas of conflict between clinical practice guidelines could allow researchers to recognise topics that have poorly been studied and focus on specific areas where the evidence is still inconclusive. In this context, a European effort is underway through the Optimal Treatment for Patients with Solid Tumours in Europe Through Artificial intelligence (OPTIMA) project [2]. OPTIMA envision is to develop comprehensible and dynamic clinical practice guidelines aiming to enhance shared decision-making by clinicians and patients. Through a centralised network of European data providers and by elaborating artificial intelligence use to analyse big data and identify the decisive factors, real-time clinical decisions based on accurate and complete weighing of available knowledge are possible. Thus, every patient will have access to the most up-to-date individualised treatment and innovative therapies.

The purpose of this study is to evaluate the quality and the consistency of international and national clinical practice guidelines on PCa management, and identify any potential differences in key recommendations by using the Appraisal of Guidelines for Research & Evaluation II (AGREE II) tool.

## 2 Methods

A stakeholder engagement was carried out including urologists, oncologists, radiologists, researchers, patients, and guideline developers. Eight key stakeholders from different countries, including the UK, France, the Netherlands, Belgium, Germany, and Sweden, were involved in the evaluation of clinical practice guidelines. In addition, clinical practice guidelines were identified from the Guidelines International Network using the terms “prostate cancer”, “guidelines”, “indications”, and “official positions”, and from national urological societies. The included clinical practice guidelines were evaluated using the AGREE II tool via the official online platform. Before the appraisal, each assessor received online training and unrestricted access to training material from the AGREE II website. The AGREE II tool comprises 23 different items organised into six domains, rated on a 7-point scale (1: strongly disagree to 7: strongly agree) [3]. Each domain represents a distinct aspect of the guideline quality. Domain 1 (scope and purpose) comprises three items and assesses whether the overall objective, health question, and population to which the clinical practice guidelines are meant to be applied are described specifically. Domain 2 (stakeholder involvement) comprises three items and assesses whether the clinical practice guideline development group includes individuals from all relevant professional groups, whether patient views and preferences have been sought, and whether end users have been defined clearly. Domain 3 (rigour of development) comprises eight items and assesses the methodology of the clinical practice guideline development to identify and present the evidence as well as to formulate the recommendations. Domain 4 (clarity of presentation) comprises three items and assesses whether the clinical practice guidelines are specific and unambiguous, whether the different management options are described clearly, and whether the key recommendations are identifiable easily. Domain 5 (applicability) comprises four items and considers the implementation in clinical practice, potential barriers, potential resource implications, and monitoring or auditing criteria. Domain 6 (editorial independence) comprises two items, and evaluates whether the editorial is independent from the funding body and the transparency of panel members in terms of conflicts of interest. Each domain score was calculated as the average score among the assessors; the total score of that domain was calculated using the following formula: (obtained score – minimal possible score)/(maximal possible score – minimal possible score) × 100 [4].

Furthermore, two additional items evaluated the global impression of the assessor. The first item “overall quality assessment” was rated from 1 to 7 (1: strongly disagree to 7: strongly agree). The second item assessed whether the clinical practice guidelines would be recommended for use in clinical practice with three prespecified answers (yes, yes—with modifications, and no).

Each clinical practice guideline was assessed by two urologists, who were native speakers of the guideline language and were familiar with it in terms of language and implementation in clinical practice, and a separate stakeholder’s group that helped identify international and national guidelines. As the clinical practice guidelines were assessed by the urologists who were native speakers, this might be perceived as a limitation due to their geographical location. . For every item, the assessors provided further comments to support their scoring. Each clinical practice guideline assessment score was arbitrated by the stakeholders to ensure fairness and conformity of the evaluation. The scores were then collated and summarised on a shared platform, and each document received a mean percentage and a mean score value. The agreement between assessors’ scores was calculated using the interclass correlation coefficient (ICC). The ICC score was defined as poor (<0.20), fair (0.21–0.40), moderate (0.41–0.60), good (0.61–0.80), and very good (0.81–1.00) [5].

As per the AGREE II protocol, there is no overall score that can be used to recommend for or against the use of a specific clinical practice guideline. However, several previous studies have ranked clinical practice guidelines as of high (five to six domains scored >60%), average (three to four domains scored >60%), and poor (one to two domains scored >60%) quality for implementation into clinical practice [6], [7], [8].

Furthermore, with regard to the quality evaluation using AGREE II, we assessed the concordance in different recommendations between clinical practice guidelines. Clinicians were asked to provide a list of key recommendations involving screening, investigation, treatment, and follow-up for patients with PCa, focusing on known discrepancies between clinical practice guidelines. A consensus meeting between clinicians and stakeholders identified the most important (key) recommendations for further evaluation. These are as follows: (1) recommendations on PCa screening, (2) recommendations on active surveillance (AS) inclusion criteria, (3) recommendations on the use of positron emission tomography (PET)/computed tomography (CT) in PCa, and (4) recommendations on the use of androgen deprivation therapy (ADT) and androgen receptor–targeted agents (ARTAs).

The primary endpoint of the study was to identify high-quality clinical practice guidelines based on the AGREE II tool. The secondary endpoints included the agreement between assessors and to recognise the inconsistencies in prespecified key recommendations. The descriptive and statistical analyses were conducted using Microsoft Excel. The *AGREE II Handbook* including the tool is available at the following link: https://www.agreetrust.org/wp-content/uploads/2013/10/AGREE-II-Users-Manual-and-23-item-Instrument_2009_UPDATE_2013.pdf.

## 3 Results

### 3.1 Guideline selection

Clinical practice guidelines from 11 national and international societies were identified (Table 1). Three clinical practice guidelines (by de l'Association Française d'Urologie [AFU], American Society of Clinical Oncology [ASCO], and AUA) have published separate documents for specific disease stages, which were evaluated independently. The assessment of the 16 clinical practice guidelines with the use of the AGREE II tool is presented in Table 2.

**Table 1:** Key clinical practice guidelines selected for the study

Guidelines	
AFU	de l'Association Française d'Urologie
ASCO	American Society of Clinical Oncology: 1. Clinically localised prostate cancer 2. Noncastrate advanced, recurrent, or metastatic prostate cancer 3. Systemic therapy update on Lu-PSMA for metastatic castration-resistant prostate cancer
AUA	American Urological Association: 1. Early detection of prostate cancer: AUA/SUO guideline (2023) 2. Clinically localised prostate cancer: AUA/ASTRO/SUO guideline (2022) 3. Advanced prostate cancer: AUA/ASTRO/SUO guideline
EAU	European Association of Urology
ESMO	European Society for Medical Oncology
NVU	Nederlandse Vereniging voor Urologie
KCE	Federaal Kenniscentrum - Centre fédérald'expertise (Belgian Health Care Knowledge Centre): 1. A national clinical practice guideline on the management of localised prostate cancer (part 1) 2. A national clinical practice guideline on the management of localised prostate cancer (part 2)
NCCN	National Comprehensive Cancer Network
NICE	National Institute for Health and Care Excellence (England, UK)
NPV	Prostatacancer - Nationellt vårdprogram (Sweden)
S3LL PCa (DE)	Interdisziplinäre Leitlinie der Qualität S3 zur Früherkennung, Diagnose und Therapie der verschiedenen Stadien des Prostatakarzinoms

ASTRO = American Society for Radiation Oncology; PSMA = prostate-specific membrane antigen; SUO = Society of Urologic Oncology.

**Table 2:** Summary of average reviewer AGREE II scores for all guidelines assessed

Name of the guideline	AFU guidelines 2021 and 2022		ASCO guidelines 2021 and 2022			AUA 2020 and 2022			EAU 2023	ESMO 2020 and 2023	NVU2014	NICE 2021	S3LL PCa	KCE 2014	NCCN 2023	NPV 2022
									All stages	All stages	All stages	All stages	All stages	All stages	All stages	All stages
Stage	L	M	L	NCA	S-CPPC	E	L	A								
Domain 1: scope and purpose (%)	69	69	97	83	67	83	78	89	92	67	64	81	100	95	43	89
Domain 2: stakeholder involvement (%)	81	64	81	75	19	69	69	89	81	42	31	78	89	89	84	78
Domain 3: rigour of development (%)	82	80	70	90	38	83	94	86	96	47	46	82	94	82	61	90
Domain 4: clarity of presentation (%)	89	94	89	94	61	86	97	97	100	83	53	89	89	87	94	89
Domain 5: applicability (%)	71	52	21	88	29	56	25	60	58	46	13	60	67	29	100	88
Domain 6: editorial independence (%)	75	83	100	100	92	96	100	83	92	92	54	63	25	71	100	83
Total score, mean (%) (SD)	77.8 (7.5)	73.7 (15)	76.3 (29)	88.3 (8.7)	51 (27.3)	78.8 (14.1)	77.2 (28.3)	84 (12.6)	86.5 (15.3)	62.8 (21.2)	43.5 (18.5)	75.5 (11.5)	77.3 (28)	75.5 (24.2)	80.3 (23.4)	86.2 (4.7)
Scoring (max 7)	6	6	4	6.5	3	6.5	6	6	6.5	5	3.5	6	6.5	6.5	6.5	6.5
Overall assessment 1 (%)	83	83	50	92	33	92	83	83	92	67	42	83	83	83	83	92
Overall assessment 2 a	I: 1 II: 2	I: 1 II: 2	I: 2 II: 3	I: 1 II: 1	I: 3 II: 3	I: 1 II:1	I: 1 II: 1	I: 1 II: 1	I: 1 II: 1	I: 1 II: 2	I: 2 II: 3	I: 1 II: 1	I: 1 I: 2	I: 2 II: 1	I: 1 I: 1	I: 1 I: 1
Overall quality of the guideline	High	High	High	High	Average	High	High	High	High	Average	Low	High	High	High	High	High

A = advanced; AFU = de l'Association Française d'Urologie; AGREE II = Appraisal of Guidelines for Research & Evaluation II; ASCO = American Society of Clinical Oncology; AUA = American Urological Association; E = early detection; EAU = European Association of Urology; ESMO = European Society for Medical Oncology; KCE = Belgian Health Care Knowledge Centre; L = localised; M = metastatic; NCA = noncastrate advanced; NCCN = National Comprehensive Cancer Network; NICE = National Institute for Health and Care Excellence; NPV = Prostatacancer - Nationellt vårdprogram; NVU = Nederlandse Vereniging voor Urologie; PCa = prostate cancer; S-CPPC = systemic CPPC; SD = standard deviation.

a Overall assessment 2: I and II stands for appraisers 1 and 2, respectively. Scoring: 1: yes, I would recommend this guideline; 2: yes, I would recommend this guideline with modifications; 3: no, I would not recommend this guideline.

### 3.2 Quality assessment

Overall, the quality of the clinical practice guidelines on PCa management is considered high, because most of the documents scored above 60% in at least five domains. The clinical practice guidelines with the highest overall scores were those of the EAU, S3LL PCa, Belgian Health Care Knowledge Centre (KCE), National Comprehensive Cancer Network (NCCN), and Prostatacancer—Nationellt vårdprogram (NPV), and the early detection of PCa and noncastrated advanced (NCA), recurrent or metastatic PCa guidelines of the AUA received the highest overall scores (6.5/7), with a mean domain score of 82.4% (range: 75.5–88.3%). Both AFU and AUA, as well as NICE received an overall appraisal score of 6/7, with a mean domain score of 77.6% (range: 73.7–84.0%). Below average were the European Society for Medical Oncology (ESMO), localised and S-CPPC ASCO, and Nederlandse Vereniging voor Urologie (NVU) guidelines, with a mean score of 3.8/7 and a mean domain score of 58.4% (range: 43.5–76.3%). The ICC ranges from 0.58 to 0.84, with an average of 0.72 (standard deviation: 0.08). The highest variability was observed in the NVU and the lowest in the AFU-L guidelines.

The evaluation of distinct clinical practice guidelines using the AGREE II scoring criteria reveals varying differences within the domains of the guideline quality assessment. Domain scores ranged from 13% (NVU, “applicability” domain) to 100%. When examining the scores of each domain, “clarity of presentation” (domain 4) achieved the highest score, with a mean of 86.9% ± 12.6%. The applicability domain (domain 5) received the lowest score, with a mean of 48.3% ± 24.8%.

The mean score of “scope and purpose” (domain 1) was 79.1% (±15.1%). The S3LL PCa, ASCO-L, KCE, and EAU clinical practice guidelines achieved a score of above 90%, while NCCN had the lowest score (43%). The mean score of the “stakeholder involvement” domain (domain 2) was 69.9% (±21.2%), with the AUA-A, S3LL PCa, and KCE clinical practice guidelines achieving the best scores (89%), while the ASCO-S-CPPC and NVU scores were 19% and 31%, respectively. The mean score of “rigour of development” (domain 3) was 76.3% (±18.5%). Four clinical practice guidelines scored above 90% (ASCO noncastrated advanced [NCA], AUA-L, EAU, S3LL PCa, and NPV), while ESMO and NVU guidelines were below 50%. In the clarity of presentation domain (domain 4), the ASCO-NCA, AUA-L, AUA-A, EAU, and NCCN clinical practice guidelines scored above 90% and NVU had the lowest value (53%). In the applicability domain (domain 5), the NCCN, NPV, and ASCO NCA had the highest scores—100, 83, and 100, respectively. In contrast, the lowest score was achieved by the NVU (13%) and ASCO-L (21%). The “editorial Independence” domain (domain 6) was scored highly across most guidelines except for the NVU and S3LL PCa, which scored significantly lower (54% and 25%, respectively). Only for the ASCO-S-CPPC guidelines, the assessors have recommended against its use.

### 3.3 Analysis of key recommendations

#### 3.3.1 Key recommendation 1: consistency of recommendations on PCa screening

All the guidelines recommend against prostate-specific antigen (PSA) screening in asymptomatic men aged under 40 yr or with life expectancy of <10 yr, except the AFU guidelines, which reduce this period to <5 yr. PSA and digital rectal examination testing are mostly advised to be offered to well-informed men aged >50 yr willing to undergo PCa screening. The AUA clinical practice guidelines concludes that men aged 55–69 yr would benefit more from screening. An individualised risk-adapted strategy is suggested for younger patients, and most guidelines recommend starting screening at age of >45 yr for patients with a family history of PCa and those of African descent. Some guidelines (AFU, EAU, ESMO, NCCN, and NPV PCa) also take into consideration the mutation status of the patient and recommend starting screening at the age of 40 yr for BRCA1/2 or HOXB13 mutation carriers. As for follow-up screening frequency, most clinical practice guidelines agree that this should be individualised based on the initial PSA and patient age. Some clinical practice guidelines such as those of the EAU suggests to postpone follow-up PSA measurements in those not at risk for up to 8 yr and stop PSA testing for men older than 70 yr.

#### 3.3.2 Key recommendation 2: consistency of recommendations on AS inclusion criteria

Many clinical practice guidelines recommend that patients with PSA <10 ng/dl are suitable for AS. The NCCN clinical practice guidelines also include men with PSA 10–20 ng/dl if <50% of biopsy cores were positive. As for pathological staging, all clinical practice guidelines advocate AS in patients with Gleason score (GS) 3 + 3 (International Society of Urological Pathology [ISUP] 1); however, three clinical practice guidelines recommend AS in men with GS 3 + 4 (ISUP 2) if additional criteria are met, such as low-volume disease (ASCO-L), PSA <10 ng/dl and stage <T2b (NCCN), and involvement <10%, PSA <10 ng/dl, and stage <cT2a (EAU). The latter excludes AS patients with intraductal or cribriform patterns despite PSA or GS. The strength of the recommendations for AS was reported in five clinical practice guidelines (EAU, ESMO, KCE, NCCN, and S3LL PCa). The various thresholds that are used as inclusion criteria for AS by the clinical practice guidelines are summarised in Table 3.

**Table 3:** Thresholds used by the 11 guidelines to advise on active surveillance

	PSA (ng/ml)	Pathology	T stage	Patient characteristics	Other thresholds used for inclusion	Recommendation strength
AFU	≤10	GG 1	T1c-T2a	LE >10 yr		NR
ASCO	NR	GS ≤6	Localised prostate cancer	LE >5 yr	Age <75 yr	NR
		GS 3 + 4 = 7 a			Age >75 yr	
AUA	<10	Grade group 1 (GS 3 + 3)	T1-T2a		Systematic biopsy with ultrasound or MRI-guided imaging	NR
EAU	<10	GS 3 + 3 (GG 1)	cT1-cT2a	LE >10 yr	No intraductal or cribriform histology on biopsy	Strong
		Highly selected patients with GS 3 + 4 (GG 2) b				Weak
ESMO	≤10	GS ≤6	T1-T2a			II A
NVU	<10	GS <7	T1c-T2a		≤2 positive cores	NR
KCE	<10	GS <7	T1-T2a	Fit for radical treatment	Age 50–80 yr	Strong
NCCN	<10	GG 1	T1c	LE ≥20 yr	PSA density and core positivity c	II A
			T1-T2a	LE ≥ 10 yr	–	
	10–20	GG 1 or 2	T2b-T2c	LE ≥ 10 yr	1 IRF d <50% biopsy core positive	
NICE	<10	GS ≤6	T1-T2a	Suitable for radical treatment		NR
NPV	≤10	GS ≤6	T1-T2a		Systematic biopsy with ultrasound or MRI-guided imaging	NR
S3LL PCa	<10	GS 6	cT1-cT2a		*Inclusion criteria*: ISUP 1 and low-risk group according D’Amico ISUP 2 and favourable risk (ISUP 2, no intraductal or cribriform PCa, Gleason grade 4 ≤10% *Exclusion criteria for AS*: PSA >15 ng/ml ISUP 2 and Gleason grade 4 >10% ISUP ≥3 cT3, cN+	4 A

AFU = de l'Association Française d'Urologie; ASCO = American Society of Clinical Oncology; AUA = American Urological Association; EAU = European Association of Urology; ESMO = European Society for Medical Oncology; GG = grade group; GS = Gleason score; IRF = intermediate risk factor; ISUP = International Society of Urological Pathology; KCE = Belgian Health Care Knowledge Centre; LE = life expectancy; MRI = magnetic resonance imaging; NCCN = National Comprehensive Cancer Network; NICE = National Institute for Health and Care Excellence; NPV = Prostatacancer - Nationellt vårdprogram; NR = not reported; NVU = Nederlandse Vereniging voor Urologie; PCa = prostate cancer; PSA = prostate-specific antigen.

a With low-volume Gleason pattern 4 (not further defined).

b That is, <10% pattern 4, PSA <10 ng/ml, <cT2a, low disease extent on imaging and biopsy.

c Fewer than three prostate biopsy fragments/cores positive, ≤50% cancer in each fragment/core, PSA density <0.15 ng/ml/g.

d 1 IRF: PSA 10–20 ng/ml or GG 2 or T2b-T2c.

#### 3.3.3 Key recommendation 3: consistency of recommendations on the use of PET/CT in PCa management

Several clinical practice guidelines have recommended the use of prostate-specific membrane antigen (PSMA)-PET/CT in biochemical recurrence after local therapy. The use of PSMA-PET in high-risk patients has been recommended by the S3LL PCa and NPV as a weak recommendation. The NPV clinical practice guidelines recommended its use in staging imaging to assess oligometastatic disease if it can guide decision-making. The EAU clinical practice guidelines recommend the use of PSMA-PET/CT in intermediate-risk disease (weak recommendation) and in high-risk localised disease/locally advanced disease (strong recommendation) if this imaging modality is available. [9], [10].

#### 3.3.4 Key recommendation 4: consistency of recommendations on the use of ADT and ARTAs

The clinical practice guidelines summarised the use of ADT in two distinct conditions: first, as a curative intent in combination with external beam radiotherapy for intermediate-risk (6 mo; AFU, ASCO, AUA, ESMO, NPV, S3LL PCa, Danish, and KCE) and high-risk localised and locally advanced (24–36 mo; AFU, ASCO, AUA, ESMO, NPV,S3LL PCa, Danish, and KCE) PCa, and second, as a palliative measure for metastatic PCa (AFU, ASCO, AUA, Danish, EAU, ESMO, NCCN, NICE, NPV, and S3LL PCa) along with ARTAs. The ASCO clinical practice guidelines recommend the use of ADT with abiraterone plus prednisone after radiotherapy in locally advanced hormone-sensitive disease. A summary of ARTA + ADT recommendations is outlined in Table 4.

**Table 4:** Summary of the ARTA + ADT recommendations according to guidelines

	EAU-EANM-ESTRO-ESUR-ISUP-SIOG guidelines	AUA guidelines	ASCO guidelines	ESMO guidelines	NCCN guidelines
Locally advanced disease	NA	NA	ADT ± abiraterone ± prednisolone for men with noncastrate locally advanced nonmetastatic prostate cancer who have undergone radiotherapy, rather than castration monotherapy	NA	NA
First-line treatment of metastatic disease	ADT + abiraterone acetate + prednisone or apalutamide/enzalutamide to patients whose first presentation is M1 disease and who are fit enough for the regimen (Strength rating: strong)	ADT ± androgen pathway directed therapy (abiraterone acetate plus prednisone, apalutamide, enzalutamide) or chemotherapy (docetaxel) for mHSPC(Strong recommendation; evidence level: grade A)	ADT + docetaxel, abiraterone, enzalutamide, or apalutamide should be offered to men with metastatic noncastrate prostate cancer, including those who received prior therapies, but have not yet progressed	ADT for mHNPC + abiraterone/prednisone (ESMO-MCBS v1.1 score: 4) or apalutamide (ESMO-MCBS v1.1 score: 4) or docetaxel (ESMO-MCBS v1.1 score: 4) or enzalutamide (ESMO-MCBS v1.1 score: 4)	Novel ARTAs are an option in M1 castration-naïve disease with ADT
Nonmetastatic castrate-resistant disease	Apalutamide, darolutamide, or enzalutamide to patients with M0 CRPC and a high risk of developing metastasis (PSADT <10 mo) to prolong time to metastases and overall survival (Strength rating: strong)	Apalutamide, darolutamide, or enzalutamide with continued ADT to nmCRPC patients at high risk for developing metastatic disease (PSADT ≤10 mo) (Strong recommendation; evidence level: grade A)	NA	Apalutamide (ESMO-MCBS v1.1 score: 3), darolutamide (ESMO-MCBS v1.1 score: 3), or enzalutamide (ESMO- MCBS v1.1 score: 3) for M0 (on bone scan and CT) CRPC and a high risk of disease progression	Enzalutamide, apalutamide, or darolutamide in M0 CRPC patients
Systemic treatment of castrate-resistant disease	Abiraterone, cabazitaxel, docetaxel, enzalutamide, olaparib, radium-223, sipuleucel-T Following docetaxel chemotherapy for mCRPC—abiraterone, cabazitaxel, enzalutamide, radium-223, and olaparib in case of DNA homologous recombination repair alterations Offer abiraterone or enzalutamide to patients previously treated with one or two lines of chemotherapy (Strength rating: strong)	In newly diagnosed mCRPC, ADT + abiraterone acetate + prednisone, docetaxel, or enzalutamide (Strong recommendation; evidence level: grade A [abiraterone acetate plus prednisone and enzalutamide]/B [docetaxel])	NA	Abiraterone or enzalutamide (ESMO-MCBS v1.1 scores: 4) recommended for asymptomatic/mildly symptomatic men with ChT-naive mCRPC In patients with mCRPC in the postdocetaxel setting, abiraterone (ESMO-MCBS v1.1 score: 4), enzalutamide (ESMO-MCBS v1.1 score: 4), and cabazitaxel (ESMO-MCBS v1.1 score: 3) are recommended options (I, A)	Abiraterone or enzalutamide should be considered in mCRPC disease

ADT = androgen deprivation therapy; ARTA = androgen receptor–targeted agent; ASCO = American Society of Clinical Oncology; AUA = American Urological Association; ChT = chemotherapy; CRPC = castration-resistant prostate cancer; CT = computed tomography; EANM = European Association of Nuclear Medicine; EAU = European Association of Urology; ESMO = European Society for Medical Oncology; ESMO-MCBS = ESMO Magnitude of Clinical Benefit Scale; ESTRO = European Society for Radiotherapy and Oncology; ESUR = European Society of Urogenital Radiology; ISUP = International Society of Urological Pathology; mCRPC = metastatic castration-resistant PCa; mHNPC = metastatic hormone-naïve PCa; mHSPC = metastatic hormone-sensitive PCa; NA = not available; NCCN = National Comprehensive Cancer Network; nmCRPC = nonmetastatic castration-resistant PCa; PCa = prostate cancer; PSADT = prostate-specific antigen doubling time; SIOG = International Society of Geriatric Oncology.

*Dependent on performance status, symptoms, comorbidities, location, extent of disease, genomic profile, patient preference, and previous treatment for metastatic hormone-sensitive PCa.

In men with metastatic hormone-sensitive PCa, the EAU and NCCN recommend the use of ADT plus ARTAs as first-line treatment, while the AUA, ASCO, and ESMO recommend chemotherapy as an additional option. In men with nonmetastatic castration-resistant PCa, the EAU, AUA, ESMO, and NCCN recommend the use of ADT plus ARTAs. Finally, in men with metastatic castration-resistant PCa, the EAU, AUA, NCCN, and ESMO clinical practice guidelines recommend the use of abiraterone or enzalutamide as an alternative to other systemic treatments such as chemotherapy; however, the choice of treatment should be based on performance status, symptoms, comorbidities, and previous therapies.

The majority of clinical practice guidelines (AFU, EAU, AUA, NCCN, ESMO, and NPV) also highlighted screening requirements before ADT administration, including HbA1c, serum lipid levels, vitamin D, calcium, osteoporosis, and fracture risk. Additionally, the EAU recommends a cardiology consultation in older men with cardiovascular disease. Men receiving ADT could be offered zoledronic acid (first intention) or denosumab (ESMO, NCCN, NICE, and NPV), and should be advised on modifying lifestyle (diet, exercise, smoking cessation, adequate calcium and vitamin D status, etc.; AFU, EAU, NICE, AFU, NCCN, and NPV).

## 4 Discussion

### 4.1 Summary of the key findings

In this study, several comprehensive national and international clinical practice guidelines for PCa have been identified, which serve as a valuable clinical resource in the management of these patients. Our main finding is that most of the clinical practice guidelines on PCa management are of high quality. The AGREE II tool is well known and used commonly for evaluating the quality and rigour of the clinical practice guidelines. By systematically assessing the six domains, the tool can identify the top-quality clinical practice guidelines that offer reliable recommendations for enhancing patient care and decision-making in PCa management. However, it is crucial to recognise that AGREE II is an appraisal tool, and not a guarantee of clinical validity or usefulness of guidelines. Therefore, clinical judgement should always be exercised when applying guideline recommendations in individual patient care.

### 4.2 AGREE II scores

The domains with higher scores were clarity of presentation (domain 4) and editorial independence, scoring 87% and 80.9%, respectively. The performance of the former was anticipated since it is a fundamental part of the recommendation process that cannot be omitted. This finding is in accordance with previous guideline appraisals regardless of the topic [7]. The latter exceeded expectations because clinical practice guideline developers are autonomous and ought not to be influenced by any external factors such as financial, commercial, or other conflicts of interest that could potentially bias the content, integrity, or recommendations of the guidelines. It is noteworthy that all major international clinical practice guidelines such as those of the EAU, ASCO, AUA, ESMO, and NCCN scored above 90%. This indicates that the clinical practice guidelines for PCa are reliable and trustworthy, and these can effectively inform health care decision-making without being influenced by external interests.

The applicability domain achieved the lowest score at 54%. The ASCO, AUA, and NVU clinical practice guidelines were among those with a very low score of <30%. It refers to the consideration of factors such as feasibility, resource implications, and potential barriers to implementing the recommendations in real-world clinical settings. Overall, the low applicability score suggests that the clinical practice guidelines may face challenges in being implemented effectively in clinical practice due to factors such as unclear recommendations, limited stakeholder involvement, etc.

The Inter-rater variability was considered good given the mean ICC of 0.72.

### 4.3 Evaluation of key recommendations

The concordance of the clinical practice guidelines was evaluated using four predefined key recommendations, and the agreement was considered good. Screening for PCa was the first key recommendation under evaluation. Most clinical practice guidelines recommend PSA screening based on age or other risk factors. Some clinical practice guidelines recommend PSA screening in known positivity for BRCA1/2 or HOXB13 mutations. The second key recommendation was the AS criteria, which also showed considerable agreement. However, since the evidence in AS is evolving, new definitions and threshold criteria are emerging regarding the upper limit of inclusion for AS (eg, GS 3 + 4, more than three positive cores, >50% core involvement, PSA cut-off, and life expectancy) [11]. The third key recommendation was the use of PET/CT, which is currently receiving a lot of attention. The utility of PET/CT in the management of PCa has been shown in several clinical contexts, including its use in high-risk PCa, in those with metastatic disease for PCa staging, in those with biochemical recurrence after radical prostatectomy, and in patients with oligometastatic disease. However, there is no clear evidence as to which patients would benefit from its use and at which stage of their disease. Clinical trials have shown promising results, offering hope that these will provide a more sensitive measure of disease spread and an alternative to lymph node biopsy [12]. In those where metastatic disease is confirmed by PSMA-PET/CT, it should be considered that treatment recommendations in all clinical practice guidelines are based on CT and bone scans. The fourth key recommendation was the use of ADT and ARTAs in PCa management. Even though there is agreement regarding the addition of ADT to adjuvant radiotherapy, there are differences in the use of ARTAs, such as recommendations for advanced localised disease (ASCO) and metastatic hormone-sensitive disease.

When clinicians practise in accordance with high-quality clinical practice guidelines, there is improvement in patient outcomes such as survival rates, quality of life (QoL), and treatment efficacy [13]. A particular aspect in this improvement is the involvement of patient representatives within the guideline panels. Including patients even from the initial phases of the clinical practice guideline development process results in the identification of important primary clinical issues and broadens the scope of the clinical practice guidelines from a patient’s perspective, thus showing more emphasis on patient involvement and shared decision-making [14]. This role has been shown to result in significant benefits for patients and is at the forefront of medical research. Bryant et al in their systematic review emphasised the importance of adding patient perspective in developing clinical practice guidelines. Despite literature highlighting its importance, this study demonstrated that there is no consensus on how patient representatives were chosen, recruited, and involved in the clinical practice guideline panels. The EVOLVE framework provides guidance on how to meaningfully engage patients in the clinical practice guideline development process [15]. Highly scoring clinical practice guidelines such as those of the NICE, EAU, NVU, and NPV have placed important considerations on patient perspective; yet, further improvement is needed in other guidelines such as those of the NCCN and ESMO. The EVOLVE framework can help clinical practice guideline developers to engage patients meaningfully in their guideline development process [15].

An excellent example of patient involvement was demonstrated recently by the PIONEER Consortium, an international multistakeholder collaboration lead by the EAU, which involved patient representatives in developing a research agenda and a core outcome set, and in the identification and prioritisation of the most relevant research questions in the field of PCa [16], [17]. By involving patients who have been through the process, guideline panels and large research collaboratives such as PIONEER are able to identify and target key areas that are of importance to patients due to their effect on QoL measures.

The guidelines that obtained high score in the AGREE II assessment emphasised the significance of QoL as a crucial outcome measure following any therapeutic intervention. Guidelines such as those by the EAU have dedicated a whole section discussing the QoL in patients with localised or metastatic PCa [18]. This is shown to be especially important when assessing the impact of a treatment in palliative settings where QoL is the main priority for patients and their families, and this is also highlighted by the other major guidelines such as those of the AUA, AFU, and NICE. The KCE also highlighted randomised controlled trials with QoL as their primary outcomes when comparing radical prostatectomy with watchful waiting (the SPCG4 and UMEA-1 trials). Interestingly, the NVU looks at QoL in terms of symptoms experienced by patients compared with population. It subdivides the symptoms, mainly exhaustion, sexual dysfunction, and libido, to highlight the treatment-specific impact. In contrast, other clinical practice guidelines such as those by the ASCO, NCCN, and ESMO report neither on QoL nor on the impact of treatment on patients. The ASCO clinical practice guidelines mention the harm versus benefit ratio; however, the quantification is unclear.

Our comprehensive analysis revealed a consensus among most guidelines regarding the role of the innovative approaches in PCa. The relevance of these approaches was expounded, with consideration given to current literature across the majority of guidelines. Discrepancies identified in clinical practice guidelines could be attributed to various factors, such as the last time the clinical practice guidelines were updated and the incorporation of the latest pertinent literature, particularly in rapidly evolving areas of research for novel approaches. Additionally, the divergent perspectives observed in guidelines may stem from the influence of national clinical practices and variations in treatment approaches influenced by patient perspectives and expectations, and health economics in different countries. The aetiology of observed differences remains speculative, and the complexity of contributing factors underscores the need for cautious interpretation. Notably, the credibility and applicability of varying treatment recommendations in clinical practice hinge on the robustness and quality of the supporting clinical practice guidelines.

AGREE II, despite being one of the most popular and well-established guideline appraisal tools used worldwide, is not devoid of limitations [19]. Furthermore, each assessor reviewed the clinical practice guidelines that they use in their everyday clinical practice. Although this allowed for better interpretation of the guidelines, an element of reporting bias may have been introduced. A way to avoid this would have been to carry out a double-blinded assessment for scoring each clinical practice guideline. We nonetheless minimised this by discussing the scores given to each guideline as a group, paying particular attention to extreme scores. We also reviewed collectively the justifications that assessors gave for their scores. The last limitation to our study was that the selection of assessors was influenced by availability and interest in the project, meaning that a large proportion of our assessors had been or were actively involved in the OPTIMA project at the time of selection. However, as they adhered to a standardised tool for reporting, prior or current involvement in the study should have only a minimal impact, if any, on their appraisals.

## 5 Conclusions

As far as we are aware, this is the first study in which a comprehensive quality assessment of the majority of international and national clinical practice guidelines was undertaken, and the key recommendations were compared to assess consistency. Our study shows that the majority of international and national clinical practice guidelines demonstrate high-quality standards when assessed using the AGREE II evaluation tool. The clinical practice guidelines that did not meet the expected standards could be improved by adopting several key recommendations outlined by our study. Regarding the level of discrepancy between guidelines, we have found that most clinical practice guidelines agreed on the core aspects of PCa management and evaluated the same body of evidence to elaborate their guidelines. Where divergences arose from out of date or erroneous evidence, these should imperatively be resolved. However, total agreement is not to be pursued per se as these could arise from different, though equally valid, interpretations of existing evidence and the need to adapt to the peculiarities of each national health system.

  ***Author contributions*:** Muhammad Imran Omar had full access to all the data in the study and takes responsibility for the integrity of the data and the accuracy of the data analysis.

  *Study concept and design*: N’Dow, Omar.

*Acquisition of data*: Sakalis, Bhattacharya, Beyer, Murray, Smith, Willemse, Gandaglia, Boissier, Borkowetz, Dabestani, Leenen, Vilaseca, Maresca, Teoh, Rivas, Rajwa, Lardas, Grivas, Van den Broeck, Pradere, Schouten, Tandogdu, Omar.

*Analysis and interpretation of data*: Sakalis, Omar.

*Drafting of the manuscript*: Sakalis, Bhattacharya, Willemse, Gandaglia, Boissier, Borkowetz, Dabestani, Omar.

*Critical revision of the manuscript for important intellectual content*: Sakalis, Bhattacharya, Beyer, Murray, Smith, Willemse, Gandaglia, Boissier, Borkowetz, Dabestani, Leenen, Vilaseca, Maresca, Teoh, Rivas, Rajwa, Lardas, Grivas, Van den Broeck, Pradere, Schouten, Tandogdu, Evans-Axelsson, Maclennan, Thomas, Briganti, Bjartell, Cornford, Kruger, N’Dow, Roobol, Omar.

*Statistical analysis*: Sakalis, Omar.

*Obtaining funding*: OPTIMA Consortium.

*Administrative, technical, or material support*: Bhattacharya, Beyer, Murray, Smith, Omar.

*Supervision*: Omar.

*Other*: None.

  ***Financial disclosures:*** Muhammad Imran Omar certifies that all conflicts of interest, including specific financial interests and relationships and affiliations relevant to the subject matter or materials discussed in the manuscript (eg, employment/affiliation, grants or funding, consultancies, honoraria, stock ownership or options, expert testimony, royalties, or patents filed, received, or pending), are the following: Angelika Borkowetz is an expert and mandate holder for the S3LL. Emma Jane Smith is the manager for the EAU Guidelines Office. Susan Evans-Axelsson is an employee of Bayer AG, which makes products used to treat PCa. Muhammad Imran Omar is the Vice Chair of the EAU Guidelines Office Methods Committee, which can be perceived as a potential conflict of interest.

  ***Funding/Support and role of the sponsor*:** OPTIMA is funded through the IMI2 Joint Undertaking and is listed under grant agreement no. 101034347. IMI2 receives support from the European Union’s Horizon 2020 research and innovation programme and the European Federation of Pharmaceutical Industries and Associations (EFPIA). IMI supports collaborative research projects and builds networks of industrial and academic experts in order to boost pharmaceutical innovation in Europe. The views communicated within are those of OPTIMA. Neither the IMI, nor the European Union, EFPIA, or any associated partners are responsible for any use that may be made of the information contained herein.

  ***Acknowledgements*:** The OPTIMA Consortium include the following members: James N'Dow, Wim Witjes, Emma Jane Smith, Carla Bezuidenhout, Sarah Collen, Karin Plass, Torsten Gerriet Blum, Angelika Borkowetz, Peter-Paul Willemse, Philip Cornford, Saeed Dabestani, Maurice Schlief, Juan Gómez Rivas, Anders Bjartell, Monique Roobol, Katharina Beyer, Lionne Venderbos, Sebastiaan Remmers, Daan Nieboer, Raoul Boomsma, Teun Kampman, Bertrand De Meulder, Charles Auffray, Nesrine Taibi, Ayman Hijazy, Albert Saporta, Johann Pellet, Muhammad Imran Omar, Lesley Anderson, Steven MacLennan, Sara MacLennan, Valerie Speirs, Solveiga Zibaite, Moragh Boyle, Charlotte Murray, Diane Brown, Demi McDonald, Andres Metspalu, Jaak Vilo, Raivo Kolde, Sulev Reisberg, Elena Sügis, Marek Oja, Telver Objartel, Alberto Briganti, Giorgio Gandaglia, Martina Faticoni, Greta Matteuzzi, Claude Chelala, Louise Jones, Maryam Abdollahyan, Emanuela Gadaleta, Guido Juckeland, Michael Bussmann, Daniel Kotik, Artur Yakimovich, Torsten Bauer, Jens Kollmeier, Jessica Werchan, Torsten Blum, Rebecca Graebig-Rancourt, Tobias Sjöblom, Chatarina Larsson, Arvid Widenlou Nordmark, Daniel Prieto-Alhambra, Sara Khalid, Edward Burn, Antonella Delmestri, Mahkameh Mafi, Danielle Newby, Cheryl Tan, Nikolaus Forgó, Antoni Napieralski, Martina Wimmer, Katharina Haimbuchner, Saskia Kaltenbrunner, Katja Hartl, Kseniia Guliaeva, Giuseppe Curigliano, Carmen Criscitiello, Stefania Morganti, Chiara Corti, Elena Dal Zotto, Nadia Harbeck, Julian Koch, Neal Navani, Sam Janes, Amyn Bhamani, Stephane Lejeune, Mario Campone, Jean-Sebastien Frenel, Kevin Joubel, François Bocquet, Camille Berneur, Marion Laloue, Malvina Dutot, Ludovic Jacob, Delphine Macle, Stéphanie Thauvin, Fanny Seguin, Catherine Le Manach, Philippe Lambin, Anshu Ankolekar, Talita Duarte-Salles, Laura Perez, Valérie Vaccaro, Thomy Tonia, Céline Genton, Wouter van Geffen, Ilona Tietzova, Armin Frille, Vincent Fallet, Adrien Costantini, Simone Wesselmann, Christoph Kowalski, Nora Tabea Sibert, Ellen Griesshammer, Pippa Powell, Clare Williams, Jessica Denning, Sigrid van Dorp, Nadia Honing, Javier Téllez, Sandra Garrido, Roberto Galán, Ruben Villoria, Inmaculada Perea Fernández, Paloma López de Arenosa Barbeito, Enric Bousoño Borrull, Laura Tur Giménez, Soralys Hernandez, Pablo Gonzalez Fuente, Juan Miguel Auñón García, José Carlos Barrios González, Alvaro Morandeira Galban, Andreas Kremer, Maria Quaranta, Sebastiano La Ferla, Loic Marc, Nils Christian, Christian Bauer, Mariana Pina, Sigrid Auweter, Julia Reichwald, Corinna Zur Bonsen-Thomas, Larissa Tschetsch, Francisco Pinto, Samuel Lesuffleur, Matthieu Blottière, Louise Duflot, David Vallas, Pierre-Olivier Chaudé, Marie Baumier, Daniele Cremonini, Patrizia Torremante, Florian Fromm, Verena von Scharfenberg, Karin Rosenits, Nuno Azevedo, Marcel Hartig, Waltraud Kantz, Frederic Kube, Amanda Matthews, Bhakti Arondekar, Bruno Gori, Hagen Krüger, Julia Ilinares, Keith Wilner, Lucile Serfass, Lynn McRoy, Robert Miller, Simon Bauer, Sofia Simon, Georgios Papanastasiou, Karen Godbold, Edwina Cahill, Stefan Langhammer, Anne Adams, Sebastian Boie, Florian Reis, Susan Evans Axelsson, John-Edward Butler-Ransohoff, Imke Meyer, Selmin Ulusu Saatci, Samu Kurki, Helene Ostojic, Abdelali Majdi, Santiago Villalba, Sai Jasti, Adrian Wolny, Lisa Schneider, Adrian Rousset, Ivo Cleuren, Sandra Eketorp Sylvan, Ellie Paintin, Monika Pokrzepa, Carolin Lorber, Marlene Thomas, Stefanie Morris, Joao Mouta, Martina von Meyenn, Mahesh Shivhare, Thomas Metcalfe, Camille Andre, Tobias Schulte in den Baeumen, Jason Hannon, Alan Mark Hochberg, Kartick Sukumaran, Jie Shen, Nareen Katta, Devanchi Patel, Yilin Xu, Sean Turner, John Ossyra, David Dellamonica, Heather Moses, Yiduo Zhang, Christophe Dufour, Marcus Simon, Maria Teresa Campos, Hassan Naqvi, Jens Ceder, Olga Alekseeva, Burkhard Mueller, Tobias Flosdorf, Ruben Koch, Anastasia Goette, Gustaf Hedström, and Per-Henrik Edqvist.
